# Framingham risk score for estimation of 10-years of cardiovascular diseases risk in patients with metabolic syndrome

**DOI:** 10.1186/s41043-017-0114-0

**Published:** 2017-11-13

**Authors:** Leila Jahangiry, Mahdieh Abbasalizad Farhangi, Fatemeh Rezaei

**Affiliations:** 1 Tabriz Health Services Management Research Center, Health Education and Health Promotion Department, Tabriz University of Medical Sciences, Tabriz, Iran; 2 Drug Applied Research Center, Department of Community Nutrition, Faculty of Nutrition, Tabriz University of Medical Sciences, Tabriz, Iran; 3 Department of Social Medicine, School of Medicine, Jahrom University of Medical Sciences, Jahrom, Iran

**Keywords:** Framingham risk score, Metabolic syndrome, Cardiovascular disease

## Abstract

**Background:**

There are a few studies evaluating the predictive value of Framingham risk score (FRS) for cardiovascular disease (CVD) risk assessment in patients with metabolic syndrome in Iran. Because of the emerging high prevalence of CVD among Iranian population, it is important to predict its risk among populations with potential predictive tools. Therefore, the aim of the current study is to evaluate the FRS and its determinants in patients with metabolic syndrome.

**Methods:**

In the current cross-sectional study, 160 patients with metabolic syndrome diagnosed according to the National Cholesterol Education Adult Treatment Panel (ATP) III criteria were enrolled. The FRS was calculated using a computer program by a previously suggested algorithm.

**Results:**

Totally, 77.5, 16.3, and 6.3% of patients with metabolic syndrome were at low, intermediate, and high risk of CVD according to FRS categorization. The highest prevalence of all of metabolic syndrome components were in low CVD risk according to the FRS grouping (*P* < 0.05), while the lowest prevalence of these components was in high CVD risk group (*P* < 0.05). According to multiple logistic regression analysis, high systolic blood pressure (SBP) and fasting serum glucose (FSG) were potent determinants of intermediate and high risk CVD risk of FRS scoring compared with low risk group (*P* < 0.05).

**Conclusion:**

In the current study, significant associations between components of metabolic syndrome and different FRS categorization among patients with metabolic syndrome were identified. High SBP and FSG were associated with meaningfully increased risk of CVD compared with other parameters.

**Trial registrations:**

The study is not a trial; the registration number is not applicable.

## Background

Metabolic syndrome defined as a cluster of metabolic risk factors including hypertension, obesity, hyperglycemia, and central obesity [1] is one of the most common health problems throughout the world. The disease has a strong association with the risk of cardiovascular events. It has been estimated that men with metabolic syndrome have 2–3 times greater probability for cardiovascular disease (CVD) than those without metabolic syndrome [2–4]. Overall, metabolic syndrome is associated with a twofold increase in the risk of CVD, CVD mortality, and stroke, and a 1.5-fold increase in risk of all-cause mortality [5–7]. In fact, coronary artery disease (CAD) is among the main causes of death in developed countries, while it has a growing epidemic in developing countries [8, 9]. The Framingham risk score (FRS) is a simplified and common tool for the assessment of risk level of CAD over 10 years [10]. The FRS considers six coronary risk factors, including age, gender, total cholesterol (TC), high density lipoprotein cholesterol (HDL), smoking habits, and systolic blood pressure [11]. FRS is the most applicable method for predicting the person’s chance of developing cardiovascular disease (CVD) in long term [12]. Because this risk score gives an indication of the likely benefits of prevention, it can be useful for both the patients and clinicians deciding whether lifestyle modification and preventive medical treatment and for patients education by identifying men and women at increased risk for future cardiovascular events [13]. Since metabolic syndrome is a complete cluster of metabolic risk factors of cardiovascular events including insulin resistance, central obesity, diabetes mellitus, and hyperlipidemia [14], it is necessary to predict the risk of cardiovascular disease in these patients. Moreover, limited data are available evaluating the predictive value of FRS in detecting the risk of CVD in patients with metabolic syndrome, and because of difference in the nature of CVD risk factors in different populations, its replication seems to be necessary [13]; therefore, the aim of the current study is to evaluate the CVD risk factors in Iranian patients with metabolic syndrome using FRS.

## Methods

### Subjects

This study is a part of the “Red Ruby” study, a cross-sectional investigation of 160 Iranian adult patients with metabolic syndrome living in Tehran; the details of this study has been reported elsewhere [15, 16]. Briefly, this was an internet-based lifestyle modification interactive program, and we enrolled community-dwelling individuals from June 22 to August 22, 2012. Target group of the study was the patients with metabolic syndrome according to the National Cholesterol Education Program’s Adult Treatment Panel (NCEP-ATP)-III criteria [17] (except for waist circumference which was defined as ≥ 90 cm for both genders for Iranian population [18]). Participants were aged 20 years and above and have no history of diabetes, cancer, and cardiovascular and/or renal diseases. They also did not have any history of taking medication for hypertension or dyslipidemia. The protocol of the current study has been approved by the ethics committee of Tehran University of Medical Sciences (97/130/1736) and ethics committee of Tabriz University of Medical Sciences (5/92/1228), and written informed consent was obtained from all of the participant’s prior participation in the study.

### Clinical and laboratory assessments

Anthropometric assessments and blood pressure measurements were measured by trained nurses as described previously [16]. Biochemical parameters including serum total cholesterol TC, low density lipoprotein cholesterol (LDL-C), high density lipoprotein cholesterol (HDL-C), fasting serum glucose (FSG), and triglyceride (TG) were measured by enzymatic colorimetric method (Pars–Azmoon, Tehran–Iran).

### Assessment of the cardiovascular risk

FRS was used to investigate the risk of cardiovascular disease [11]. FRS scores were calculated based on the six coronary risk factors including age, gender, TC, HDL-cholesterol, systolic blood pressure, and smoking habits. The cutoffs for calculating FRS were as follows: TC < 160, 160–199, 200–239, 240–279, and ≥ 280 mg/dL; for systolic blood pressure: < 120, 120–129, 130–139, 140–159, and ≥ 160 mmHg; and for HDL-C: < 40, 40–49, 50–59, and ≥ 60 mg/dL [11]. Ten-year risk in percentage was calculated by total points (1 point 6%, 2 points 8%, 3 points 10%, 4 points 12%, 5 points 16%, 6 points 20%, 7 points 25%, 10 points or more > 30%). Absolute CVD risk percentage over 10 years was classified as low risk (< 10%), intermediate risk (10–20%), and high risk (> 20%) [11].

### Statistical analysis

Statistical analysis was performed with Statistical Package for Social Science (SPSS 18 for windows, SPSS Inc.® headquarter, Chicago, USA). Normality of data was analyzed by Kolmogorov-Smirnov test. Continuous and discrete variables are presented as mean ± SD and number and percent, respectively. Analysis of variance (ANOVA) was used to test the difference between biochemical variables between three groups. Multiple logistic regression analysis was used to examine the association between risk factors of metabolic syndrome and 10-year risk for cardiovascular disorders (according to FRS scoring) as independent and dependent variables, respectively. Adjusted odds ratio and 95% confidence intervals were calculated for FRS different levels. The potential confounding variables in the multiple logistic regression analysis were age and body mass index. *P* values less than 0.05 were regarded as statistically significant.

The sample size of the patient population was calculated based on the overall prevalence of MS in the Iranian general population [19]. An estimate of that magnitude with 95% confidence limits and 80% relative precision required a sample size of 140. With a 10% allowance for any dropouts, the calculated sample size was 160.

## Results

**Fig. 1:**
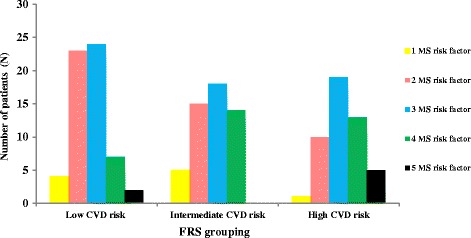
Association between Framingham risk score risk categories and number of metabolic syndrome components

The mean age of participants was 44.02 ± 10.02 years old. Overall, 77.5, 16.3, and 6.3% of patients with metabolic syndrome were at low, intermediate, and high risk of CVD according to FRS categorization. Baseline characteristics of study participants are presented in Table 1. As shown in this table, mean age of participants was 44.02 ± 10.02, and 33.8% of participants were female gender. Figure 1 presents the association between number of metabolic syndrome components and FRS categories among participants. As shown in this figure, the highest prevalence of patients with two or three risk factors of metabolic syndrome were in low and intermediate risk of CVD, while the patients with four or five risk factors of metabolic syndrome were most prevalent in groups with high CVD risk. Prevalence of metabolic syndrome components in different categories of FRS is presented in Table 2. The highest prevalence of all of metabolic syndrome components was in the low-CVD risk according to the FRS grouping (*P* < 0.05), while the lowest prevalence of these components was in the high CVD risk group (*P* < 0.05).

**Table 1:** Baseline characteristics of patients with metabolic syndrome

Characteristics	*N* = 160
Age (year)	44.02 ± 10.02
Female gender (%)	54 (33.8%)
SBP (mmHg)	131.78 ± 11.03
DBP(mmHg)	88.33 ± 6.45
WC (cm)	104.47 ± 8.76
FSG (mg/dl)	90.09 ± 13.89
Serum TG (mg/dl)	193.81 ± 113.02
Serum TC (mg/dl)	195.28 ± 32.84
Serum HDL (mg/dl)	41.26 ± 9.97

*SBP*, systolic blood pressure; *DBP*, diastolic blood pressure; *WC*, waist circumference; *FSG*, fasting serum glucose; *TG*, triglyceride; *TC*, total cholesterol; *HDL*, high density lipoprotein

**Table 2:** Prevalence of metabolic syndrome components according to Framingham risk score (FRS)

	*N* (%)	Low risk *N* (%)	Intermediate risk *N* (%)	High risk *N* (%)	P*
Patients in group	160 (100)	124 (77.5)	26 (16.3)	10 (6.3)	–
High SBP (mmHg)	125 (78.1)	90 (72)	25 (20)	10(8)	*0.007*
High DBP (mmHg)	140 (87.5)	112 (80)	22 (15.7)	6 (4.3)	*0.04*
High WC (cm)	118 (73.8)	93 (78.8)	19 (16.1)	6 (5.1)	*0.018*
High FSG (mg/dl)	16 (10)	7 (43.8)	6 (37.5)	3 (18.8)	*0.002*
High Serum TG (mg/dl)	107 (66.9)	81 (75.7)	17 (15.9)	9 (8.4)	0.276
Low Serum HDL (mg/dl)	114 (71.3)	87 (76.3)	19 (16.7)	8 (7)	0.582

*SBP*, systolic blood pressure; *DBP*, diastolic blood pressure; *WC*, waist circumference; *FSG*, fasting serum glucose; *TG*, triglyceride; *HDL*, high density lipoprotein concentrations. Italic digits provide significant values

*Chi-square test was used for comparisons

Multiple logistic regression evaluating the association between FRS risk categories and risk factors of metabolic syndrome (Table 3) showed that high blood pressure and fasting serum glucose were potent determinants of intermediate and high risk categories of FRS compared with the low-risk group (*P* < 0.01, *P* < 0.05, respectively).

**Table 3:** Association between risk factors of metabolic syndrome and 10-year risk for cardiovascular disorders (according to Framingham risk score (FRS) scoring)

Parameter		FRS		
		Low (< 10%)	Intermediate (10–20%)	High (> 20%)
Age (year)	OR	Ref.	1.30	1.82
	95% CI	1	1.17–1.45	1.38–2.39
Female gender	OR	Ref.	0.057	0.068
	95% CI	1	0.014–0.242	0.017–0.089
High SBP (mmHg)	OR	Ref.	*4.98*	*5.70*
	95% CI	1	*2.3–59.12*	*3.12–10.15*
High DBP (mmHg)	OR	Ref.	0.25	0.18
	95% CI	1	0.04–1.77	0.009–3.66
High WC (cm)	OR	Ref.	1.67	3.55
	95% CI	1	0.4–6.36	0.27–45.76
High FSG (mg/dl)	OR	Ref.	*3.7*	*4.07–71.55*
	95% CI	1	*1.16–7.45*	*1.25–47.14*
High TG (mg/dl)	OR	Ref.	2.06	0.58–7.28
	95% CI	1	14.35	1.75–19.13
Low HDL (mg/dl)	OR	Ref.	0.59	0.17–2.00
	95% CI	1	0.25	0.019–3.4

*Ref*, reference group; *SBP*, systolic blood pressure; *DBP*, diastolic blood pressure; *WC*, waist circumference; *FSG*, fasting serum glucose; *TG*, triglyceride; *HDL*, high density lipoprotein concentrations. Italic digits provide significant values

## Discussion

In the present study, there was a significant relationship between FRS risk scores and components of metabolic syndrome including high systolic blood pressure (SBP), diastolic blood pressure (DBP), waist circumference (WC), and FSG. Moreover, according to the results of logistic regression analysis, high SBP and high FSG make patients 3–5 times more susceptible to be in intermediate and high risk of cardiovascular disease. These findings were in accordance of the results of previous studies. In the study by Takahashi et al. [8], CAD risk was associated with higher SBP, TC, and lower HDL concentrations in general population. In their study, having metabolic syndrome presented a fourfold greater probability of high CAD risk score. Other study by Yousefzadeh et al. [13] also found higher prevalence of 10-year risk of CVD in patients with metabolic syndrome (*P* < 0.001).

Totally, 77.5% of patients were in low risk of CVD, whereas 16.3 and 6.3% were in intermediate and high risk of CVD, respectively. These values were comparable with the previous study performed in Kerman, Iran, while the corresponding numbers were 74.3, 18.1, and 7.6%, respectively. Moreover, in our study, the number of metabolic syndrome components in patients of high risk score was significantly higher than the other groups. Therefore, FRS can be used as a diagnostic tool for presence of metabolic syndrome as also confirmed by previous studies [5, 10, 13]. In the study by Takahashi et al. [8], same as our results, a positive correlation was observed between CAD risk score and the number of metabolic syndrome components; the greater the metabolic syndrome components, the higher the risk of developing CAD, although the results of studies in the predictive capacity of FRS in cardiovascular disease risk are inconsistent. Several studies reported that metabolic syndrome is a better predictor of CVD risk compared with FRS because of high dependency of FRS to age, underestimation of cardiovascular disorders in young ages, and lack of coverage several prominent features of metabolic syndrome such as obesity, hyper-triglyceridemia, and elevated high CRP levels [20, 21]; however, two previous US reports [22, 23] showed that FRS is more predictive for CVD risk than metabolic syndrome. More studies in different age groups and geographical locations are needed to address this question.

Although the FRS is a useful tool for predicting the risk of CVD, however, it has also several limitations that should be considered before applying its results to population: first of all, the FRS is an estimation algorithm and cannot be used as a medical examination; secondly, because of the under-representation of young population in the original cohort, the FRS may be an imprecise tool in this population; and thirdly, the FRS did not include several other potential CVD risk factors like family history of CVD or diabetes [24, 25]. Moreover, the cross-sectional nature of the current study makes it difficult to better address the direction of causation between variables.

## Conclusion

The current study, for the first time, evaluated the predictive value of FRS for estimation of 10-year CVD risk in patients with metabolic syndrome in Iran. Due to relatively high prevalence of metabolic syndrome in Iranian population, the results will be useful for designing interventional strategies to prevent CVD in these patients. In the current study, a potent relationship between FRS risk scores and components of metabolic syndrome has been identified. High SBP and FSG were associated with meaningfully increased risk of CVD compared with other parameters. More studies are needed to further clarify these associations in a longitudinal design.

## Abbreviations

ANOVA: Analysis of variance
CAD: Coronary artery disease
CRP: C-reactive protein
CVD: Cardiovascular disease
DBP: Diastolic blood pressure
FRS: Framingham risk score
FSG: Fasting serum glucose
HDL: High density lipoprotein
LDL: Low density lipoprotein
NCEP-ATP: National Cholesterol Education Adult Treatment Panel
SBP: Systolic blood pressure
TC: Total cholesterol
TG: Triglyceride
WC: Waist circumference
